# Benzodiazepine Misuse: An Epidemic Within a Pandemic

**DOI:** 10.7759/cureus.15816

**Published:** 2021-06-21

**Authors:** Ashish Sarangi, Terry McMahon, Jayasudha Gude

**Affiliations:** 1 Psychiatry, Texas Tech University Health Sciences Center, Lubbock, USA; 2 Psychiatry, Zuckerside Hillside Hospital, New York, USA

**Keywords:** benzodiazepine, covid-19, pandemic, coronavirus, psychiatry

## Abstract

Coronavirus disease 2019 (COVID-19) had deleterious effects on patients with mental health problems and several studies have shown a spike in the rates of depression, insomnia, and post-traumatic stress disorder. Anxiety and insomnia rates have also increased among both the general public and health care professionals. Benzodiazepines are some of the most commonly used drugs in the treatment of anxiety and insomnia. However, benzodiazepines are also misused, abused alone, or abused in combination with other drugs. Lockdowns and social distancing have also had negative consequences on patients with mental health problems. We assessed the extent of benzodiazepine use during the pandemic and interpreted its effects in the future. We conducted a literature search using the Preferred Reporting Items for Systematic Reviews and Meta-Analyses (PRISMA) protocol and eight articles reviewed specifically reported worrying fluctuations in benzodiazepine use during the pandemic. We observed varied trends in the usage of benzodiazepines in various parts of the world. Some studies showed an increase in the consumption of benzodiazepine while others demonstrated a decrease in the prescription refills of benzodiazepine, which may be a result of gaps in mental health care. At this time, we can conclude that the current trend with benzodiazepine use is fluctuating and mental health professionals must continue to exercise caution before prescribing benzodiazepines. Future research is also warranted to be aware of the changing patterns and to avoid misuse and/or abuse at an epidemic level.

## Introduction and background

It has been more than a year since the coronavirus disease 2019 (COVID-19) was first reported in December of 2019 [1]. According to World Health Organization, there have been 170 million cases reported and almost every country has been affected by the coronavirus pandemic [1,2]. The COVID-19 outbreak has led people everywhere to implement drastic changes in lifestyle, including social distancing. Periods of social isolation and loneliness have resulted in negative consequences on mental well-being. It was found that people were three times more likely to have anxiety or depressive disorders in 2020 compared to the previous year [3], and more than one in three individuals presented one or both disorders. According to the CDC, from January 20, 2021, to February 1, 2021, more than two in five adults aged ≥18 years experienced symptoms of anxiety or a depressive disorder during the past seven days [4]. It has also been reported that symptom rates of generalized anxiety, psychological distress, and COVID-19 related fear are 44.9%, 65.2%, and 59% respectively [5]. Since the pandemic onset, there has been also a 37% increase in the rate of clinical insomnia (from 14.6% to 20%) [6]. Among health care workers caring for COVID-19 patients, there has been an increase in the prevalence of anxiety to 25.8% (95% CI 20.5-31.9%) and stress to 45% (95% CI 24.3-67.5%) [7].

Benzodiazepines are a class of medications used to treat conditions such as anxiety and insomnia. In 1955, the first benzodiazepine (BZD), chlordiazepoxide, was developed, followed by diazepam in 1963 [8-11]. In 1957, as an alternative to previous barbiturates, BZD use started to gradually replace the opiate derivates [12]. Benzodiazepines became widely used drugs because of their potential benefits and were the most abused drugs by the 1970s [13]. With growing concern about the abuse of BZDs, they were placed on Food and Drug Administration drug list [14]. The risk of BZD dependence was officially recognized by the American Psychiatric Association in 1990 [15,16]. BZDs act by binding to the gamma-aminobutyric acid A (GABA-A) [17] receptors in the brain, inhibiting the brainstem arousal pathways. When BZDs interact with GABA-A receptors, ion channels open more frequently, increasing the inflow of chloride ions which increases membrane polarization and inhibits neuron firing resulting in central nervous system (CNS) depression [15]. This mechanism results in anxiolytic, sedative, hypnotic, anticonvulsant and skeletal muscle-relaxing effects.

Benzodiazepines are classified based on their elimination half-life. They include long-acting diazepam, chlordiazepoxide, flurazepam, and clorazepate along with intermediate-acting alprazolam, clonazepam, lorazepam, oxazepam, and temazepam with short-acting agents being midazolam and triazolam. BZDs are metabolized oxidatively in the liver by the cytochrome P450 enzymes (phase I), conjugated with glucuronide (phase II), and excreted almost entirely in the urine [18]. They are prescribed for a wide range of conditions, which include insomnia, agitation, anxiety and convulsions [19]. The dose-related side effects include amnesia and central respiratory depression [18]. Benzodiazepines used in large quantities can also result in a dopamine rush, which is responsible for creating a sense of pleasure and reward [20]. Benzodiazepines are commonly consumed orally but some smoke, snort or inject. They are commonly referred to as “candy”, “downers”, “sleeping pills”, or “tranks” [21]. Flunitrazepam is one of the commonly abused drugs and has been referred to as date rape drug, roofies, or forget-me-pill [22].

Over the past several years there have been rising concerns about the misuse of benzodiazepines. A study has estimated that 30.6 million adults (12.6%) reported benzodiazepine use in the year 2015-2016 [23]. Among them they report that 2.2% have misused a BZD prescription. Among adults and adolescents, benzodiazepines are the third most commonly misused illicit or prescription drug in the USA [24,25]. Over the past few decades, benzodiazepine use has increased tremendously. A study done by Bachhuber et al. showed that the number of adults who filled a benzodiazepine prescription increased by 67%, from 8.1 million to 13.5 million between the years 1996 and 2013. The quantity obtained also increased from 1.1 kg to 3.6 kg lorazepam-equivalents per 100,000 adults [26].

According to the National Institute of Drug Abuse (NIDA), among 2017-2018 benzodiazepine misusers, 46.3% of respondents reported that the motivation for their most recent misuse was to relax or relieve tension, followed by helping with sleep in 22.4%. About 5.7% reported “experimentation” as their main motivation for misuse, and 11.8% reported using them to “get high” or because of being “hooked”. The data also showed that most misusers obtained benzodiazepines from friends or relatives, with only about 20% receiving them from their doctor [27]. Opioids have been largely involved in deaths related to overdose, and NIDA reports that benzodiazepines have been involved in more than 30% of opioid-related overdose deaths [27]. Benzodiazepines have also been implicated in causing addiction and long-term cognitive problems. NIDA also reported that among benzodiazepine users, 17.1% misused them and fewer than 2% had benzodiazepine use disorder [27]. Benzodiazepine derivatives are prescribed in large quantities globally and are potentially new emerging environmental contaminants. Several studies have emerged showing the presence of BZD derivatives in sewage water and hospital effluents [28-32]. Wastewater samples in Mississippi, USA, have been found to contain alprazolam, α-OH-alprazolam, nordiazepam, oxazepam and temazepam [32]. Scientists in Sweden found that fish living downstream from sewage treatment plants (STP) had elevated benzodiazepine levels and found concentrations of a common benzodiazepine, oxazepam, of 0.73 mg/liter in treated wastewater effluent and 0.58 mg/liter in a midsized stream (River Fyris) receiving input of treated wastewater. These studies indicate that wild fish populations may be affected, and we are still unaware of how these trace levels affect humans [33]. These studies have not only provided information on the prevalence of drug use on a community level, but also warn caution on the use of benzodiazepines.

We hypothesized an increase in benzodiazepine use during the COVID-19 pandemic and evaluated the extent of benzodiazepine use during the COVID-19 pandemic in terms of benzodiazepine prescription and other illicit use. 

Table 1 below demonstrates half-life, onset of action and dose equivalency of commonly utilized benzodiazepines.

**Table 1:** Table demonstrating half life, onset of action and dose equivalency when compared to 5mg of diazepam [17,34-38]

Benzodiazepine	Half Life in hours	Peak time of action in hrs	Dose equivalent to 5mg Diazepam
Alprazolam	12	1-2	0.5-1
Chlordiazepoxide	100	1-4	10-25
Clonazepam	18-50	1-2	0.25-0.5
Clorazepate	100	0.5-2	7.5–15
Diazepam	100	1-2	5-10
Flurazepam	100	0.5-1	15–30
Lorazepam	15	1-4	1-2
Oxazepam	8	1-4	15-30
Quazepam	25-41	1.5	10-20
Temazepam	11	2-3	10-20
Triazolam	2	1-2	0.25-0.5

## Review

Methods

We conducted an extensive literature review using databases PubMed, CINAHL, Cochrane, and Google Scholar (sorted by date and articles with abstracts only were included to review) using the keywords “benzodiazepines” and “COVID-19 Pandemic” following the Preferred Reporting Items for Systematic Review and Meta-Analysis (PRISMA) protocol [39]. Inclusion criteria included research conducted in all countries after the beginning of the coronavirus pandemic, and both English and non-English articles were included. Studies that reported the use of benzodiazepine since the beginning of the pandemic were included. Excluded studies include non-peer-reviewed publications, case reports, case series, review articles, and letters to editors. Studies involving substance abuse but not mentioning specifically benzodiazepine use were also excluded. We included articles from January 1, 2020, to April 30, 2021.

**Figure 1:**
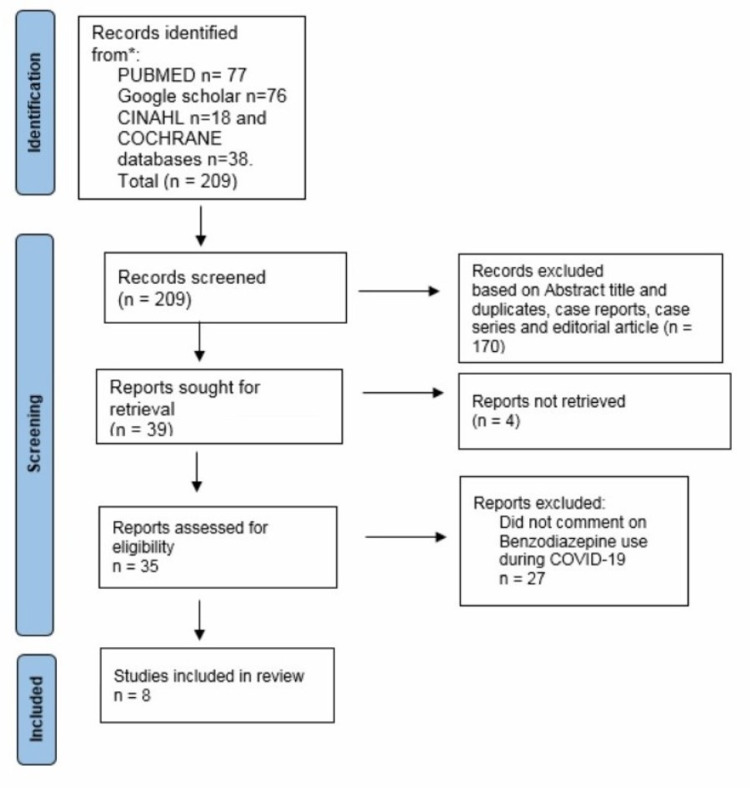
Preferred Reporting Items for Systematic Review and Meta-Analysis (PRISMA) flow diagram depicting the process of identification and selection of articles.

Figure 1 below depicts the process of review and inclusion of articles for the purposes of this review.

Results

Our search strategy resulted in 209 studies; 31 studies are excluded based on title screening. One hundred seventy articles were excluded for various reasons. Reasons for exclusion included duplicates articles (35), case reports (26), case series (seven), review articles (61), and editorial articles (10). Following this, 39 articles were retrieved out of which four were not accessible. Final full texts of 35 article are reviewed and eight studies written in English which reported the use of benzodiazepines during COVID-19 pandemic are included. The final eight articles were deemed suitable to be included for the conciseness of this review. 

Discussion

Benzodiazepines, especially diazepam, have been the main course of treatment for anxiety and insomnia [40]. Prescribing benzodiazepines has been controversial due to the recognized deleterious effects of long-term treatment with these drugs. Benzodiazepine use in COVID-19 patients has been shown to exacerbate delirium and suppress respiratory drive in patients with respiratory suppression [41]. Caution has been exercised in the use of benzodiazepines, especially palliative care. Some drugs such as midazolam and triazolam are contraindicated with the use of lopinavir/ritonavir because of the risk of increasing the level of some benzodiazepines due to CYP450 inhibition [41]. With the emerging coronavirus pandemic, most countries have updated their drug prescription policies [1]. In the USA, Prescription Drug Monitoring Program (PDMP) is an electronic way to monitor the dispensing of controlled substances used in most states [42].

The COVID-19 pandemic has caused a disruption of the availability of critical mental health services and as a result many people may have faced an increase in the use of alcohol and drugs [1]. Benzodiazepines may have a high potential for abuse and misuse during the pandemic [43], and they are typically co-abused in patients with substance use disorders [44]. The most frequent primary abuse drugs are opioids and/or alcohol; benzodiazepines are misused to enhance the other drug’s euphoric effects, reduce the unwanted effects of drugs, such as insomnia due to stimulant use, and alleviate withdrawal symptoms between doses [45]. According to a recent study, among 196 participants, 47% reported an increase in substance use since COVID-19 related to loss of employment, fear of catching the virus, social distancing and isolation. Some people reported substance use as a coping mechanism to deal with the negative physical, mental and social impacts of COVID-19 [46].

In Dublin, measures have been taken promptly after the declaration of the COVID-19 pandemic by the WHO. There was a growing concern about the spread of coronavirus and mortality among people with drug dependency and homelessness. Sixty-two percent of homeless people on opioid substitution therapy also misused street benzodiazepines [47,48]. So, clients were placed in isolation units and shielding units. Patients with benzodiazepine dependency were offered up to 30 mg of benzodiazepine daily to prevent withdrawals for the period of isolation only. In the homeless sector, over 70 people were commenced on benzodiazepine maintenance treatment [49]. In Ireland, it has been reported that 92% of overdoses where methadone was implicated and 81% of deaths where heroin was implicated involved benzodiazepines predominantly [47]. In France, because of social isolation or psychological troubles due to lockdown with the potential increase of marital conflicts and domestic violence, there was a prediction for increased consumption of benzodiazepines [50]. Further data collection by the French addictovigilance centers, pharmacies, and addiction specialized centers revealed abuse or misuse with alcohol or other psychoactive substances. Among benzodiazepines, clonazepam, alprazolam, and oxazepam were the most frequently reported to be misused or abused [50].

Lockdown has also resulted in a lot of online browsing about potential drugs of abuse. Google trends in health-related research showed a significant increase in online search interest for the keywords representing benzodiazepines during the lockdown in India [51]. Ireland also reported an increase in the use of alcohol and benzodiazepine use during lockdown periods which has continued after the lockdown as well [52]. The use of illicit drugs has returned to pre-lockdown levels, leading to an elevated risk of developing comorbid psychiatric disorders and other health conditions [52].

The United Nations reports that the non-medical use of benzodiazepines is a well-known phenomenon and represents an increasingly widespread public health problem during the pandemic since its magnitude is difficult to estimate, mainly due to the lack of monitoring and data collection in most countries [53]. This misuse pattern is associated with an elevated risk of serious health consequences or fatal overdose, especially among high-risk opioid users, who misuse benzodiazepines to increase the effects of opioids or to self-medicate to treat symptoms of psychiatric disorders, negative emotional states, opioid withdrawal symptoms, and the side effects of alcohol and cocaine use. The diversion of benzodiazepines most commonly involves alprazolam, diazepam, and lorazepam which seem to have a wider diffusion than oxazepam, clorazepate, and chlordiazepoxide because of their higher abuse potential [53]. In Europe, causes of deaths among high-risk opioid users are more frequently related to the use of benzodiazepines with rapid onset of action such as diazepam, clonazepam, and alprazolam rather than those with a slower onset such as oxazepam and flunitrazepam [54].

In Texas, USA, a study was done to observe the trend in benzodiazepine prescription during the pandemic. According to the Texas Prescription Monitoring Program, the average daily number of patients who filled new benzodiazepine prescriptions was 17,548.56 (Standard Deviation (SD) = 1295.06). There was a significant decrease in the benzodiazepine prescriptions between January 5, 2020, and May 12, 2020 (β = −1982, 95%CI = −3712.43, −252.14) [55]. A similar trend was observed with benzodiazepine prescribers [55]. On March 22, 2020, the governor issued an executive order restricting elective medical procedures including many routine outpatient visits. Before the executive order, there was an average of 9,087.24 (SD = 356.26) unique daily prescribers of new benzodiazepine prescriptions. There was a significant decrease in benzodiazepine prescribers associated with the executive order (β = −708.62, 95%CI = −1190.54, −226.71). This result may be due to a large gap in the care for patients having benzodiazepine prescriptions that has emerged during the pandemic [55]. A study was done by Niles et al. analyzing the deidentified urine specimen samples from the Quest Diagnostics medMATCH® method. There was a significant decrease in non-prescribed benzodiazepine use (4%, p<0.02) indicating a decrease in overall misuse during the pandemic. However, among benzodiazepine users, positivity for non-prescribed fentanyl increased by 48% (P < 0.01) [56].

In the USA, a study was done comparing the unique dispense of medications from January 2019 to May 2020 [57]. The monthly number of unique patients dispensed as benzodiazepines (mean = 4,781,043 [SD = 166,850]; range = 4,478,448 to 5,011,279) was relatively stable until March 2020. Since March 2020 the number of unique patients dispensed benzodiazepines (5,128,721) was statistically and significantly higher than forecast estimates. In March 2020, an estimated additional 450,074 (95% CI:189,999 to 710,149) unique patients were dispensed benzodiazepines, compared to forecast estimates [57]. Another study was done in Italy by analyzing hair samples to see drug use patterns during the COVID-19 pandemic. It revealed the percentage of samples positive for benzodiazepines ranged from 16.7% (5/30 cases) in the period before the lockdown to 53.3% (16/30 cases, p<0.01) during the lockdown and remained high (43.3%, 13/30 cases, p<0.01) even after the lockdown [58]. The benzodiazepines reported in this study were not prescribed and their intake indicated illicit use. The study revealed changes in the overall trend of drug intake during the considered study period; 11/30 (37%) patients switched from single-drug use in the two pre-lockdown period controls to poly-drug use in the post-lockdown period [58]. According to an Italian report during COVID-19, there has been a concerning increase in the prescription of hypnotics/sedatives with the potential for abuse, which has almost doubled and increased by about 17-19% [59,60]. In another study in Ontario, data from January 1 to May 31, 2019, were compared with data from January 1 to May 31, 2020. There was a 43.7% increase in benzodiazepine dispensing in the first five months of the year compared to the year prior [61]. The same study also reported that the number of benzodiazepine tablets dispensed monthly during the COVID-19 pandemic was statistically higher compared to the previous year (1037.4 ± 122.24, 721.6 ± 156.87, respectively, z=-2.402, p=0.016) [61].

In Spain, a telephone survey was conducted by the health professionals among the Drug Addiction Assistance of Castile and Leon (DAACYL) units. The professionals in the DAACYL units expressed that the clinical impact during the first six weeks of the pandemic was moderate; however, six centers reported that patients increased or started consuming alcohol and benzodiazepines, especially alprazolam [62]. Among people who were confirmed with coronavirus infection and discharged subsequently, there was a 3.3% initiation of new benzodiazepine prescriptions [63]. The findings of all the studies discussed are summarized in Table 2.

**Table 2:** Summary of significant findings of major studies conducted evaluating benzodiazepine use during the COVID-19 pandemic.

Author	Site of Study	Study Period	Data Collection	Sample Size	Findings
Lapeyre-Mestre et al. [50] 2020	France	March 17^th^, 2020 – May 31^st^, 2020	Data collected from French Addictovigilance Network	n=231	Significant misuse and abuse of benzodiazepines reported (with alcohol or other psychoactive substances).
Downs et al. [55] 2020	USA	January 5^th^, 2020, to May 12^th^, 2020	Texas Prescription Monitoring Program.	n=18000	Significant decrease associated with both benzodiazepine prescriptions and prescribers.
Niles et al. [56] 2020	USA	January 1^st^, 2019, through May 16^th^, 2020	De-identified results from all medMATCH specimens with clinician-provided prescribed drug information	n= 44211	There was a 4% decrease in non-prescribed benzodiazepine use (p<0.02)
Jones et al. [57] 2020	USA	January 2019-May 2020	Data from the IQVIA Total Patient Tracker database	n=5,128,721	The results of the study showed additional 450,074 (95 % CI:189,999 to 710,149) unique patients were dispensed benzodiazepines compared to forecasted estimates
Gili et al. [58] 2020	Italy	March 22^nd^, 2020 – May 18^th^, 2020	Hair Sample collected from 30 patients (aged 18–48 y; 17 males; 13 females) from urban areas of central Italy for analysis	n=30	The percentage of samples positive for benzodiazepines increased from 16.7% (5 cases) in the period before the lockdown to 53.3% (16 cases, p < 0.01) during the lockdown and remained high after the lockdown (43.3%, 13 cases, p < 0.01).
Yu et al. [61] 2020	Canada	January 1^st^ to May 31^s^t 2019 Vs January 1^st^ to May 31^st^, 2020	Prescription refill information from independent community pharmacy	n=365	There was significantly more frequent dispensing of benzodiazepine tablets (z= 2.402, p=0.016) in the first five months of 2020 compared to those of 2019.
McCarthy et al. [63] 2020	USA	March 7^th^, 2020 - March 30^th^, 2020.	Data collected from Confirmed coronavirus infection	n=213	New benzodiazepine initiation was 3.3% among discharged patients.

Five out of eight studies showed an increase in benzodiazepine use, whereas two have reported a decrease. These data indicate there have been changing trends in benzodiazepine prescription and misuse during the pandemic.

Limitations

We restricted our review to the search terms Benzodiazepines and COVID-19 pandemic, so we may have missed relevant studies which did not have these keywords. We discussed in a broader sense the use of a benzodiazepine during the pandemic including their prescriptions, rather than strictly focusing on benzodiazepine misuse. Several studies which have reported other benzodiazepine use along with opioids and other psychotropics are also included. The studies lacked clear details of benzodiazepine misuse and the individuals might have been using benzodiazepines as prescribed, not representing misuse. We also included studies done using urine and hair sample analysis rather than restricting to strict terms of benzodiazepine misuse.

## Conclusions

We discussed the various trends observed regarding the benzodiazepines use during COVID-19 pandemic as reported in different studies. We reviewed benzodiazepine misuse and predict how the trends of benzodiazepine misuse may vary in the post-pandemic era. COVID-19 has been a global pandemic that has spiked the rates of anxiety and insomnia. Mental health professionals need to be prepared for issues that may arise due to COVID-19 such as anxiety, depression, fear, coronophobia, trauma and grief due to loss of a loved one. The risk of developing benzodiazepine tolerance, abuse and addiction must be considered by health care professionals before prescribing them. This review summarizes the trends observed during the pandemic and emphasizes the importance of weighing risks versus benefits before the prescription of benzodiazepines. The studies which showed an increase in benzodiazepine use represent the tip of an iceberg. We hypothesize the trends in benzodiazepine usage may increase in the future and caution is warranted. We hope mental health professionals prescribe benzodiazepines carefully and make changes in practice according to the prescription drug monitoring program data to avoid a benzodiazepine crisis. Future research is warranted for the periodic assessments of an evolving benzodiazepine crisis.
